# A whole-joint, unidimensional, irreversible, and fine-grained MRI knee osteoarthritis severity score, based on cartilage, osteophytes and meniscus (OA-COM)

**DOI:** 10.1371/journal.pone.0258451

**Published:** 2021-10-14

**Authors:** Eric C. Sayre, Ali Guermazi, Savvas Nicolaou, John M. Esdaile, Jacek A. Kopec, Joel Singer, Hubert Wong, Anona Thorne, Jolanda Cibere

**Affiliations:** 1 Arthritis Research Canada, Vancouver, British Columbia, Canada; 2 Radiology, Boston University School of Medicine, Boston, Massachusetts, United States of America; 3 Radiology, University of British Columbia, Vancouver, British Columbia, Canada; 4 Medicine, University of British Columbia, Vancouver, British Columbia, Canada; 5 Medicine, University of Calgary, Calgary, Alberta, Canada; 6 School of Population & Public Health, University of British Columbia, Vancouver, British Columbia, Canada; Istanbul University Istanbul Faculty of Medicine: Istanbul Universitesi Istanbul Tip Fakultesi, TURKEY

## Abstract

**Objective:**

To develop a whole-joint, unidimensional, irreversible, and fine-grained MRI knee osteoarthritis (OA) severity score, based on cartilage, osteophytes and meniscus (OA-COM), and to predict progression across different severity states using OA-COM as outcome and clinical variables as predictors.

**Methods:**

Population-based knee pain cohort aged 40–79 was assessed at baseline and 7-year follow-up. OA-COM score was defined as the sum of MRI scores for cartilage, osteophytes and menisci, measured at 6, 8 and 6 sites, total score 0–54. To anchor severity levels, we fit cross-sectional logistic models using OA-COM to predict Kellgren-Lawrence (KL) grades in subsets at or one point below each grade. OA-COM threshold scores were selected on sensitivity, specificity, positive and negative predictive value. We developed longitudinal logistic models for OA-COM progression over each threshold over 7 years. Potential predictors included age, sex, BMI, malalignment, physical exam effusion, quadriceps weakness, and crepitus, selected on area under the receiver operating characteristic curve (AUC) and Akaike’s Information Criterion (AIC).

**Results:**

Optimal OA-COM thresholds were 12, 18, 24 and 30, for KL grades 1 to 4. Significant predictors of progression (depending on threshold) included physical exam effusion, malalignment and female sex, with other selected predictors age, BMI and crepitus.

**Conclusion:**

OA-COM (0–54 range) is a whole-joint, unidimensional, irreversible, and fine-grained MRI OA severity score reflecting cartilage, osteophytes and menisci. OA-COM scores 12, 18, 24 and 30 are equivalent to KL grades 1 to 4, while offering fine-grained differentiation of states between KL grades, and within pre-radiographic disease (KL = 0) or late-stage disease (KL = 4). In modeling, several clinical variables predicted progression across different states over 7 years.

## Introduction

Osteoarthritis (OA) is a highly prevalent, disabling and costly condition. In the U.S., clinical OA (defined on the basis of symptoms and physical examination findings) was seen in 27 million adults in 2008 [1]. Symptomatic radiographic knee OA affects 9.5% of elderly adults aged 63 years and older [2]. OA is more prevalent with older age and in obese people and thus, OA constitutes an increasing public health burden. In Canada, the total economic burden for OA, including direct and indirect costs, was estimated at $27.5 billion in 2010, and the cumulative economic burden between 2010 and 2040 is estimated to be $1.45 trillion [3].

Kellgren-Lawrence (KL) grade is the de facto radiographic scale for structural osteoarthritis [4]. An integer scoring system, Kellgren et al [4] describe the 5 levels of KL grade as: 0 (none), 1 (doubtful), 2 (mild), 3 (moderate), 4 (severe). Grades are based on a consideration of osteophytes, joint space narrowing (due to articular cartilage loss and/or meniscal damage/extrusion), subchondral sclerosis and subchondral cysts [4]. While for many decades the dominant scoring system for structural OA, the KL grading system has limitations. Chief amongst these is the coarse-grained property of the grading scale, accommodating 5 levels in total, with only 3 levels considered “osteoarthritis” (grades 2, 3 and 4). The primary reason for this coarse-grained property is the x-ray modality. Magnetic resonance imaging (MRI) allows for much finer discrimination of structures (including soft tissue), and as such, theoretically can provide a finer-grained scale for “OA severity”.

The purpose of this study is two-fold. First, we propose a whole-joint, unidimensional, irreversible, and fine-grained MRI OA severity score, based on summing over cartilage, osteophytes and meniscus (OA-COM). MRI scoring of joint structures has been done before [5–7], but the focus has been on individual component scoring rather than whole-joint scoring, and furthermore, dimensions have included those which are reversible (e.g., bone marrow lesions or effusion). Osteoarthritis is a whole-joint disease, and on aggregate is irreversible, and as such, a single MRI-based scale of OA severity including only components which are irreversible (analogous to the x-ray based Kellgren Lawrence grading system) could be advantageous as a research tool. To clarify, while MRI is more costly than x-ray and hence may be prohibitive in clinical practice, an MRI-based whole-joint score such as OA-COM could be used in cohort studies and/or clinical trials, to name but two possible areas of application. To further aid in the interpretation of the unidimensional OA-COM score, we used cross-sectional logistic regression to anchor four OA-COM scores at equivalent levels to KL grades 1 through 4. OA-COM thus borrows from the interpretability and familiarity advantage of the widely used KL grade, is similarly irreversible, yet offers fine-grained discrimination intra-grade that KL grade does not offer.

In the second objective, we develop longitudinal prediction models for progression at or above different OA severity anchor states in a population-based longitudinal cohort with 7-year follow-up, using OA-COM as the outcome and clinical variables as predictors.

## Materials and methods

### Ethics approval

This study was conducted in accordance with the declaration of Helsinki and was approved by the Clinical Research Ethics Board of the University of British Columbia. All participants gave written informed consent at all three time points.

### Data collection

Source data came from a longitudinal study conducted in Vancouver, Canada [8], a population-based cohort of individuals aged 40 to 79 with knee pain “on most days of the month at any time in the past and any pain in the past 12 months.” Data collection has been previously described [9,10]. The clinical examination was performed by an experienced rheumatologist (JC). We have previously reported in this cohort that, based on MRI cartilage damage and x-ray findings, 13% had no OA, 49% had pre-radiographic OA (cartilage damage but KL<2), and 38% had radiographic OA [10]. This cohort enrolled 255 individuals, stratified by age decade and sex in roughly equal group sizes to ensure adequate sample size across the age-sex spectrum [11]. Baseline visits occurred between 2002 and 2005. In addition to the baseline cycle, two follow-up cycles were undertaken, at weighted mean 3.3 (SD 0.6) and 7.5 (SD 0.6) years. The present study uses the baseline sample (N = 255), as well as the second follow-up cycle (N = 122).

The study knee was the more painful knee at baseline. X-rays were obtained using a weight-bearing fixed-flexion posteroanterior view with the SynaFlexer (BioClinica Inc., Newark, CA, USA) positioning frame, and a skyline view in the supine position [12]. Radiographs were read blinded to clinical information by two independent readers for KL 0–4 grading [4]. Previous studies using these data have demonstrated good interrater reliability (ICC = 0.79) [9]. Differences in readings were adjudicated by consensus readings with both readers. MRIs were acquired on a GE 1.5T magnet at a single centre using a transmitter-receiver extremity knee coil. The imaging protocol included four MRI sequences, as previously described [10,11]. MRIs were scored by a board-certified musculoskeletal radiologist (AG) who was blinded to clinical, radiographic, and time sequence information. Osteophytes (0: absent, 1: small, 2: moderate, 3: large) were scored in 8 regions: lateral and medial femur, lateral and medial tibia, and lateral, medial, superior and inferior patella. Cartilage was scored in 6 regions: lateral and medial femur, lateral and medial tibia, patella and trochlear groove, and graded on a 0–4 semi-quantitative scale based on the following definitions, previously described by Disler et al: [13] 0: normal, 1: abnormal signal without cartilage contour defect, 2: contour defect of < 50% cartilage thickness, 3: contour defect of 50–99% cartilage thickness, 4: 100% cartilage contour defect with subjacent bone signal abnormality. (0 and 1 were collapsed since 1 represents signal hyperintensity on T2-weighted images of indeterminate significance.) Meniscal damage was scored as: 0: normal, 1: intra-substance signal, 2: tear, 3: maceration/resection. (0 and 1 were collapsed.) Meniscal damage was scored in the following 6 regions: lateral anterior, lateral body, lateral posterior, medial anterior, medial body and medial posterior. Intra-rater reliability analyses were previously performed on the scoring of each surface within each feature. The ranges of intraclass correlation coefficients (ICCs) across regions were: osteophytes 0.77–0.89, cartilage 0.84–1.00, meniscus 0.60–0.83.

The MRI-based OA-COM score was computed as the sum of scores for cartilage, osteophytes and meniscus. The cartilage score was summed over its 6 region-specific scores each of which can range from 0–3. The osteophyte score was summed over its 8 region-specific scores each of which can range from 0–3. The meniscus score was summed over its 6 region-specific scores each of which can range from 0–2. The possible range for the OA-COM score was therefore 0–54, with higher numbers indicating worse OA disease.

Potential predictor variables for progression models all previously demonstrated good reliability according to an inter-rater intraclass correlation coefficient (ICC) of at least 0.8 [9]. Variables included age, sex, BMI, alignment by visual inspection (varus/valgus vs. normal), physical exam effusion (present/absent), crepitus (none/fine/coarse), quadriceps strength (poor/moderate, full), and KL grade (<2 vs. 2+).

### Statistical methods

To obtain population-representative results, a baseline sample weight was developed as the ratio of knee-pain population age-sex distribution over the baseline knee-pain sample distribution, and was used in the cross-sectional anchor point models. A sample weight was developed for the longitudinal sample as the ratio of baseline sample proportion in a given age-sex cell over the longitudinal sample proportion in that cell. Prediction models were weighted with the longitudinal sample weight.

For our first objective, to anchor the OA-COM score at interpretable points representing different levels of disease severity, we fit cross-sectional logistic models predicting each KL grade in a subset of data including only KL grades at or one point below the predicted grade, with the OA-COM score as predictor. For example, we predicted KL grade 3 in a subset of data including KL grades 2 and 3. The reason for fitting these models on data that included only KL grades at or one point below each predicted grade was to find thresholds with optimal discrimination at the selected KL grades. For each model, receiver operating characteristic (ROC) curves were produced, and for each cut point (i.e., OA-COM threshold score) we computed sensitivity, specificity, positive predictive value (PPV), negative predictive value (NPV), and the sum of squares (SS) of 100% minus each of those four predictive utility measures. The optimal threshold for OA-COM in predicting a given KL grade was selected from amongst the cut points with lowest SS (i.e., best balance amongst predictive utility measures), with a preference for higher PPV. Model fit was assessed via the Hosmer and Lemeshow goodness of fit test [14], as well as area under the ROC curve (AUC). Predictive utility was assessed with AUC and Akaike’s Information Criterion (AIC).

For our second objective, for each selected OA-COM cut point, we selected longitudinal logistic prediction models for progression over the given cut point from baseline to cycle 3, using the subset of data with OA-COM score under the given cut point at baseline (i.e., those who could progress). Predictors in multivariable models were selected using forward selection based on lowest AIC at each step, from aforementioned predictor variables except baseline KL grade. The primary analysis excluded KL grade because of the goal of achieving a prediction model based on clinical predictors (history and physical examination variables). As a sensitivity analysis, however, additional models were developed that included baseline KL grade as a potential predictor, and the impact on predictive utility (AUC and AIC) was assessed. We used Firth’s penalized maximum likelihood estimation in logistic models to reduce bias in the parameter estimates under small cell sizes [15].

Analyses were performed using SAS version 9.4 (SAS Institute Inc., Cary, NC, USA).

## Results

Table 1 contains the full baseline weighted sample characteristics (N = 255) on which the anchor point models were fit for our first objective, including mean (SD) of OA-COM and each of its three component sums (cartilage, meniscus, and osteophyte). 98.0 (38.4%) had radiographic OA (defined as KL grade > = 2) at baseline. OA-COM showed a clear increasing monotonic trend over KL grades 0 to 4, mean (SD) ranging from 6.8 (4.8) to 28.8 (5.4). The individual components also trended higher with increasing KL, nearly all monotonic with the exception of osteophytes which showed a plateau across KL grades 3 and 4. Cartilage sum ranged from a mean (SD) of 2.5 (2.7) to 10.6 (2.0), meniscus sum ranged from a mean (SD) of 0.3 (0.6) to 2.2 (1.0), and osteophyte sum ranged from a mean (SD) of 4.0 (2.6) to 16.3 (4.4) at KL grade 3, dipping to 16.1 (3.9) at KL grade 4.

**Table 1:** Sample characteristics in the baseline sample (N = 255): Mean (SD).

Variable	Baseline wgt. N (%)	OA-COM score	Cartilage score sum	Meniscus score sum	Osteophyte score sum
Kellgren-Lawrence grade					
0	104.9 (41.1)	6.8 (4.8)	2.5 (2.7)	0.3 (0.6)	4.0 (2.6)
1	52.1 (20.4)	11.0 (6.2)	4.5 (3.2)	0.8 (1.0)	5.8 (3.9)
2	48.7 (19.1)	17.3 (7.3)	6.8 (3.1)	1.0 (1.3)	9.6 (4.8)
3	26.9 (10.6)	28.3 (5.8)	9.9 (2.5)	2.0 (1.3)	16.3 (4.4)
4	22.3 (8.8)	28.8 (5.4)	10.6 (2.0)	2.2 (1.0)	16.1 (3.9)

Table 2 lists the baseline weighted characteristics in the longitudinal sample (N = 122) on which the OA-COM progression prediction models were fit for our second objective. Mean (SD) age was 55.5 (9.1). Mean BMI was 26.1 (4.0). Females comprised 68.0 (55.7%) of the sample. Distributions of the other predictor variables are listed in the table.

**Table 2:** Baseline sample characteristics in the follow-up sample (N = 122): N (%).

Variable	Baseline (wgt. N = 122.0)
Age, mean (SD)	55.5 (9.1)
BMI, mean (SD)	26.1 (4.0)
Female	68.0 (55.7)
Malalignment	16.1 (13.2)
Effusion	23.9 (19.6)
Quadriceps weakness	11.4 (9.4)
Crepitus	
None	37.8 (31.0)
Fine	61.4 (50.4)
Coarse	22.8 (18.7)
Radiographic OA	48.3 (39.6)

Table 3 lists the OA-COM score (and its three components) evaluated per cycle in the longitudinal sample. All four show monotonic increasing trends over time, with mean (SD) from baseline to 7-year follow-up increasing from 13.4 (8.3) to 17.8 (10.4) for OA-COM, 4.4 (3.6) to 6.3 (4.6) for cartilage sum, 1.6 (2.4) to 2.2 (2.8) for meniscus sum, 7.4 (3.8) to 9.3 (4.5) for osteophyte sum.

**Table 3:** Scores per cycle: Mean (SD).

Variable	Baseline (wgt. N = 122.0)	3-year follow-up (wgt. N = 108.6)	7-year follow-up (wgt. N = 122.0)
OACOM score	13.4 (8.3)	15.0 (8.9)	17.8 (10.4)
Cartilage score sum	4.4 (3.6)	5.1 (3.9)	6.3 (4.6)
Meniscus score sum	1.6 (2.4)	1.9 (2.8)	2.2 (2.8)
Osteophyte score sum	7.4 (3.8)	8.0 (3.9)	9.3 (4.5)

All cross-sectional logistic anchor point models passed the Hosmer and Lemeshow goodness of fit test. Table 4 lists the model AUCs, as well as selected cut points (12, 18, 24, 30) and their sensitivity, specificity, PPV and NPV. AUC for KL grades 1 to 4 were respectively 0.707, 0.767, 0.835, and 0.550, with higher KL grade models performing better generally with the exception of the KL grade 4 model. For the specific selected cut points, sensitivity ranged from 0.453 to 0.793, specificity ranged from 0.690 to 0.848, PPV ranged from 0.591 to 0.737, and NPV ranged from 0.606 to 0.857.

**Table 4:** Sensitivity, specificity, positive and negative predictive value (PPV and NPV) for selected cut points in the anchor point models.

KL grade	AUC	OA-COM score threshold	Sensitivity	Specificity	PPV	NPV
1	0.707	12	0.453	0.848	0.615	0.743
2	0.767	18	0.583	0.811	0.737	0.683
3	0.835	24	0.793	0.750	0.657	0.857
4	0.550	30	0.500	0.690	0.591	0.606

Longitudinal OA-COM progression prediction model characteristics are listed in Table 5, for sets of predictors that included/excluded baseline KL grade. Addition of the x-ray variable in sensitivity analyses did not impart a substantial improvement to the models with the exception of the highest cut point (30+) and to a lesser extent cut point 24+, for which x-ray information makes an improvement to AUC and AIC. The primary model (excluding KL grade) AUC (AIC) for cut points 12+, 18+, 24+ and 30+, are respectively 0.793 (60.4), 0.719 (96.8), 0.823 (79.0) and 0.768 (76.9). For cut points 12+ and 18+, the clinical model excluding KL grade is the best performing predictor pool, while for cut point 24+ adding x-ray improves the AUC (AIC) to 0.874 (65.0), and for cut point 30+ adding x-ray improves the AUC (AIC) to 0.907 (52.9).

**Table 5:** Predictive utility measures for selected progression prediction models.

Pools	Cut-point	Predictors	AUC	AIC
PH	12+	BMI, female*, effusion, age	0.793	60.439
PXH		BMI, female*, effusion, age	0.793	60.439
PH	18+	effusion*, BMI, female, age	0.719	96.816
PXH		effusion*, BMI, female, age	0.719	96.816
PH	24+	age, BMI, crepitus, malalignment*, effusion	0.823	78.957
PXH		radiographic OA*, BMI, age, *female*†	0.874	65.012
PH	30+	age, BMI, crepitus, *female*†	0.768	76.934
PXH		radiographic OA*, BMI, *female*†, age	0.907	52.874

* p-value<0.05.

† protective effect.

AUC = Area under the receiver operating characteristic curve.

AIC = Akaike’s Information Criterion.

All variable pools include: Female sex, age.

Physical exam (P) includes: Malalignment, effusion, quadriceps weakness, crepitus.

X-ray (X) includes: Radiographic OA.

History (H) includes: BMI.

Table 6 lists the odds ratios (ORs) and 95% confidence intervals (CIs) for the primary predictor set, for longitudinal OA-COM progression prediction models across cut points 12+, 18+, 24+ and 30+. The only significant predictor across cut point 12+ was female sex (OR = 6.82; 95% CI = 1.40, 33.33). Other selected predictors (retained due to improving predictive utility) included BMI, physical exam effusion and age. The only significant predictor across cut point 18+ was physical exam effusion (8.43; 2.20, 32.38). Other selected predictors included BMI, female sex and age. The only significant predictor across cut point 24+ was malalignment (4.68; 1.08, 20.25). Other selected predictors included age, BMI, crepitus and physical exam effusion. There were no statistically significant predictors across cut point 30+, although age was borderline significant and crepitus had a similar effect to that seen for the 24+ cut point, indicating that this may be a sample size issue. Selected predictors included age, BMI, crepitus and female sex (protective).

**Table 6:** Odds ratios (OR) for OA-COM score models in physical exam/history pool.

Cut-point	Predictor variable	OR (95% CI)
12	BMI (per unit)	1.19 (1.00, 1.41)
	Female	6.82 (1.40, 33.33)
	Effusion	8.74 (0.62, 123.53)
	Age (per 10 years)	1.34 (0.57, 3.13)
18	Effusion	8.43 (2.20, 32.38)
	BMI (per unit)	1.11 (0.99, 1.25)
	Female	2.11 (0.75, 5.94)
	Age (per 10 years)	1.13 (0.61, 2.07)
24	Age (per 10 years)	1.82 (0.99, 3.35)
	BMI (per unit)	1.03 (0.89, 1.19)
	Crepitus (fine)	0.94 (0.24, 3.64)
	Crepitus (coarse)	3.63 (0.80, 16.35)
	Malalignment	4.68 (1.08, 20.25)
	Effusion	3.56 (0.99, 12.86)
30	Age (per 10 years)	1.77 (0.99, 3.17)
	BMI (per unit)	1.07 (0.92, 1.23)
	Crepitus (fine)	4.55 (0.77, 26.86)
	Crepitus (coarse)	6.48 (0.94, 44.50)
	Female	0.51 (0.17, 1.54)

## Discussion

We have developed a whole-joint, unidimensional, irreversible, and fine-grained MRI knee OA severity score, the OA-COM score (0–54 range), which sums over multiple compartment-specific scores involving cartilage, osteophytes and menisci. The included dimensions are irreversible, and combined into one scale, which may be deemed appropriate as a measure of overall OA severity that is analogous to the pre-dominant x-ray scoring system Kellgren Lawrence grade (also based on summing over irreversible components). With 55 possible scores, OA-COM is fine-grained, yet remains anchored to the familiar Kellgren Lawrence grading system with four equally spaced OA-COM thresholds corresponding to KL grades 1, 2, 3 and 4. As osteoarthritis is both a “whole joint” disease and on aggregate is irreversible, OA-COM may be optimal for researchers familiar with the KL grading system. Specifically, that OA is a whole-joint disease is reflected in the inclusion of three components: cartilage, osteophytes and meniscus (progression in any of these components will be reflected as an increase in the overall score). Furthermore, the irreversibility of knee OA disease as a whole is reflected in the included components; notably omitted are reversible components (e.g., bone marrow lesions and effusion). To clarify its potential use as a research outcome, while MRI is more costly than x-ray and hence may be prohibitive in clinical practice, an MRI-based whole-joint score such as OA-COM could be used in cohort studies and/or clinical trials (as KL grading is currently used), to name but two possible areas of application.

Magnetic resonance imaging scoring of knee osteoarthritis has been done before [5–7], for example BLOKS (Boston Leeds Osteoarthritis Knee Score), MOAKS (MRI Osteoarthritis Knee Score), and WORMS (Whole-Organ Magnetic Resonance Imaging Score). However, each time the focus has been on individual component scoring rather than whole-joint scoring, and furthermore, components included those which are reversible (e.g., bone marrow lesions or effusion). Notably, however, osteoarthritis is a whole-joint disease, and on aggregate is irreversible, and as such, a single MRI-based scale of OA severity including only components which are irreversible (analogous to the x-ray based Kellgren Lawrence grading system) could be advantageous as a research tool. Indeed, much knee OA research has been done using KL grades (an aggregate score involving osteophytes and joint space narrowing [cartilage]), and similar lines of research would benefit from an analogous whole-joint, unidimensional, irreversible, and fine-grained scale based on MRI. It is nevertheless worth addressing some of the concerns previous papers have raised around combining different components (e.g., cartilage, osteophytes and meniscus) into a whole joint score. The first such concern raised by Peterfy et al [5] centered on relative weighting of the included dimensions (e.g., that cartilage and osteophytes would receive a relatively high weight in a simple sum score amidst other dimensions with either fewer ordinal levels or fewer summands). On the other hand, Peterfy et al themselves acknowledged that in the case of cartilage and osteophytes, that might actually be appropriate. Their other concern centered on comparability of subjects with equal total scores yet different structural defects (e.g., a knee with severe osteophytes yet moderate cartilage damage could score the same as a knee with severe cartilage damage yet moderate osteophytes). However, this may also be seen as an advantage, in that damage on different components would increase the severity score (OA after all being a whole-joint disease). Finally, it is important to note that the most longstanding and widely used unidimensional measure of structural osteoarthritis (namely Kellgren-Lawrence grade) combines different structures into one scale, and in so doing KL grade offers advantages including ease of application and interpretation for researchers, clinicians and patients. Specifically, KL grade is based most heavily on a consideration of osteophytes, along with joint space narrowing (due to articular cartilage loss and/or meniscal damage/extrusion), subchondral sclerosis and subchondral cysts, yet is represented on a single ordinal dimension [4]. Therefore it is sensible that OA-COM, anchored to the KL grading system but utilizing MRI technology, would also be represented on a single dimension. Another comparison to previous work in this area might be made with respect to the included/excluded components: notably absent from OA-COM are bone marrow lesions and effusion. As discussed above, these were not included in the OA-COM definition because they can be transient (i.e., they can be commonly reversed) unlike the included dimensions of cartilage, osteophytes and meniscal damage for which deterioration is largely irreversible. Therefore, OA-COM as defined is also analogous to KL grade with respect to monotonicity over time.

While analogous to KL grade, the fine-grained property of OA-COM offers a big advantage over the KL grading system, specifically the ability to study and discriminate amongst groups with much finer differences, and within pre-radiographic disease (KL grade 0) as well as late-stage disease (KL grade 4). OA-COM scores of 12, 18, 24 and 30 represent MRI scores that are equivalent to KL grades 1 to 4. These OA-COM thresholds are anchored to their KL grade equivalent grades with a good balance between high sensitivity, specificity, PPV and NPV, slightly favoring PPV. That the selected anchor points ended up being equally spaced was not the goal *a priori*, rather the goal in anchor point selection was to provide a good balance between sensitivity, specificity, PPV and NPV. However, the equal spacing can be considered desirable in an interval-scaling sense. Longitudinal OA-COM progression models across each threshold offer good predictive utility even when based only on a small number of easy-to-collect clinical variables: age, sex, BMI, malalignment, physical exam effusion, and crepitus. OA-COM offers the advantage of a fine-grained scoring system ranging from 0–54, representing states from no measurable OA at all at 0 (implausible in a population with knee pain, representing only 4.4% of our cohort at baseline) up to a nearly physically impossible severity level of 54 (nothing close to this was seen in our knee pain cohort which topped out at 45 at follow-up). Thus we can deduce that OA-COM cannot possibly suffer from floor effects in any conceivable population (including pre-surgical), and could only potentially suffer non-negligible ceiling effects in a non-applicable population such as a very healthy cohort without knee pain.

In longitudinal OA-COM progression modeling, we found that physical exam effusion, malalignment and female sex were significantly predictive of progression across different states of OA-COM severity over 7 years in this population-based cohort. Other selected predictors (which improved predictive utility) included age, BMI, and crepitus. These findings are consistent with studies of progression of radiographic OA (KL grade) and/or knee arthroplasty. For example, Zarringam et al reported that female sex (hazard ratio [HR] = 4.83) or higher BMI (HR = 1.08) were predictive of future knee arthroplasty among those without radiographic OA at baseline [16]. In our OA-COM 12+ model (a comparable subpopulation to Zarringam et al) these effects had similar magnitudes. In a study by Peat et al, cross-sectional predictors of radiographic OA in a subpopulation with knee pain included the majority of ours: age, male sex, BMI, physical exam effusion and crepitus [17]. Notably, the effect of sex reported by Peat et al on advanced OA is opposite Zarringam et al for early OA, and this phenomenon is also seen in our OA-COM models when comparing low to high thresholds—albeit, sex is not significant in our higher threshold model. Malalignment (the other significant predictor in our OA-COM 24+ model) has been shown to be associated with both incidence and progression of knee OA in various studies. For example, Brouwer et al [18] found associations between varus malalignment and incidence as well as progression of radiographic knee OA. Sharma et al [19] found associations between varus malalignment and incidence of knee OA, and both varus and valgus malalignment with progression of knee OA. Felson et al [20] reported associations between valgus malalignment and both incidence and progression of knee OA. Our findings on physical exam effusion are also generally consistent with the literature. For example, Roemer et al reported that baseline effusion (measured on MRI) predicted cartilage loss at 30 months in subjects without preexisting radiographic knee OA (comparable to our OA-COM 12+ and 18+ models) [21]. In a study by Wang et al, the bulge test on physical exam to assess effusion (the approach also used in our study) predicted progression of radiographic knee OA in a population with preexisting OA (comparable to our OA-COM 24+ model) [22].

It is worth noting that the addition of baseline KL grade in sensitivity analyses did not impart a substantial improvement to progression models with the exception of the highest cut point (30+) and to a lesser extent 24+, for which x-ray information made an improvement to AUC and AIC. For the 30+ model in particular, AUC improved from 76.8% to 90.7%, while AIC improved (dropped) from 76.9 to 52.9. This makes sense considering that predicting progression over the highest cut point amounts to predicting development of advanced OA, for which x-ray information would be highly relevant. Nevertheless, the AUC for the purely clinical model (without x-ray) remains viable at >75%, and that model maintains the advantage of being applicable with only a clinical examination.

The strengths and limitations of our study deserve comment. While population-based is a strength, the target population is not the general population, but those with baseline knee pain, aged 40–79 at baseline, who were followed up over an average of 7.5 years. However, considering our objective was to develop an MRI scoring system for OA (an inherently painful disease) as well as prognostic models for progression of OA-COM, this restriction should not be too impactful, and further, our inclusion of mild but persistent knee pain without diagnosed OA actually represents an *expanded* target population compared to some of the OA literature which focuses strictly on radiographic OA populations. Another limitation of the OA-COM score is that it requires an MRI, which can be expensive. However, an associated strength of this study is precisely the fact that it is based on MRI, and as such offers a wide range of applicability (from no measureable OA [OA-COM = 0] through to a nearly physically impossible degree of joint degradation [OA-COM = 54]), on a fine-grained measurement scale. Further, restriction of the OA-COM progression model predictors to clinical variables should facilitate a wide application of these prediction models in clinical settings, in addition to research application such as cohort studies and clinical trials. Finally, while being novel is a strength, it can also be considered a limitation in that OA-COM and the prediction models for it have not yet been externally validated beyond this study itself. This should be undertaken on independently collected data.

We have developed a whole-joint, unidimensional, irreversible, and fine-grained MRI knee OA severity score, the OA-COM score (range 0–54), summing over compartment scores for cartilage, osteophytes and menisci. OA-COM scores of 12, 18, 24 and 30 represent MRI scores that are equivalent (anchored to) KL grades 1 to 4, while offering fine-grained differentiation of OA states between KL grades, and within pre-radiographic disease (KL = 0) as well as late-stage disease (KL = 4). In longitudinal OA-COM progression prediction modeling, we found that physical exam effusion, malalignment and female sex were significantly predictive of progression (with other selected predictors improving predictive utility being age, BMI, and crepitus) to different states of OA-COM severity over 7 years in this population-based cohort.
